# Kimura’s disease affecting multiple body parts in a 57-year-old female patient: a case report

**DOI:** 10.1186/s13223-019-0400-z

**Published:** 2019-12-30

**Authors:** Bo Yu, Guoxing Xu, Xiaofan Liu, Wen Yin, Hao Chen, Baoqing Sun

**Affiliations:** 1 Department of Emergency, Tongji Medical College, Huazhong University of Science and Technology, The Central Hospital of Wuhan, 26 Shengli Street, Wuhan, 430014 Hubei province China; 2 Department of Allergy, Tongji Medical College, Huazhong University of Science and Technology, The Central Hospital of Wuhan, 26 Shengli Street, Wuhan, 430010 Hubei Province China; 3 Department of Allergy and Clinical Immunology, Guangzhou Institute of Respiratory Health, State Key Laboratory of Respiratory Disease, National Clinical Research Center for Respiratory Disease, Guangzhou Medical University, First Affiliated Hospital of Guangzhou Medical University, 151 Yanjiang Road, Guangzhou, 510120 Guangdong Province China

**Keywords:** Kimura’s disease, Eosinophils, Immunoglobulin E, Lymphocyte, Multiple body

## Abstract

**Background:**

Kimura’s disease (KD) is a rare chronic inflammatory disease with unknown etiology. It usually manifests as a painless soft tissue mass or subcutaneous nodule on one side of the patient’s head and/or neck and rarely affects multiple parts of the body. The disease is more common among young Asian males.

**Case presentation:**

A 57-year-old Chinese woman complained of multiple masses on her body surface. Ultrasonography was used to examine the retroperitoneal, bilateral neck, bilateral supraclavicular, bilateral axillary, and bilateral inguinal superficial lymph nodes. Enlargement of multiple lymph nodes was found in all areas. Many solid nodules were also found in the right parotid gland and right posterior neck area, respectively. Numerous solid nodules were seen on the left chest wall. Laboratory tests showed that the percentage of eosinophils in the whole blood was 39.40%, total immunoglobulin E (IgE) level was > 5000 kU/L, and serum special IgE to Phadiatop (inhaled allergens) and fx5 (food allergens) were 1.01 and 1.04 kUA/L, respectively. After a complete examination, the masses located in the right neck, retroauricular and left axillary regions, and left chest wall were resected directly. Postoperative pathological findings revealed KD.

**Conclusions:**

The case discussed in this study is extremely rare and did not meet the common affected areas and age characteristics of KD. This presentation can be used to improve disease awareness among physicians.

## Background

Kimura’s disease (KD) is a rare benign inflammatory disease with unknown etiology and limited epidemiological data. It usually manifests as a unilateral painless soft tissue mass or subcutaneous nodule in the head and/or neck, which can easily invade the salivary glands, parotid glands, and lymph nodes of the patient, occasionally accompanied by itchy skin. The main affected areas of the lesion are the head and neck [1]. Although KD is normally reported in Europe and the United States, most of the patients reported are of Asian descent. The disease is more common in young men 28 to 32 years of age and can also be found in children. Prevalence of male-to-female ratio is between 3.5:1 and 9:1 [2]. KD was first described in a Chinese study in 1937 [3], which was originally thought to be “eosinophilic proliferative lymphogranuloma”. In 1948, the definitive histological description was published by Kimura in Japan, after whom the disease was named [4]. This study aimed to report a rare case of KD in a woman with multiple-system onset accompanied by pruritus.

## Case presentation

A 57-year-old Chinese woman complained of multiple masses on her body surface for 2 years, mainly in the right cervical and retroauricular lymph nodes, with no tenderness, clear boundary, or mobility. In the absence of treatment, slow progressive enlargement and increase in numbers were noted in the masses, which were palpable on the axilla, chest wall, and groin, as well as unclear demarcation from the surrounding tissues, accompanied by itchiness of the affected skin surfaces. The patient had a medical history of tuberculosis, gastric polyp, and hysteromyoma.

Physical examination revealed two subcutaneous masses in the right posterior ear and neck measuring approximately 1 × 1 cm and 2 × 2 cm, respectively, which were solid in texture, with clear boundaries, and fixed to the skin. The skin surface was black, without tenderness, and with a rise in local skin temperature. The enlarged lymph nodes could be palpated on the chest wall and supraclavicular, axillary, and inguinal regions. Obvious scratches were found on the skin of the patient’s whole body.

**Fig. 1:**
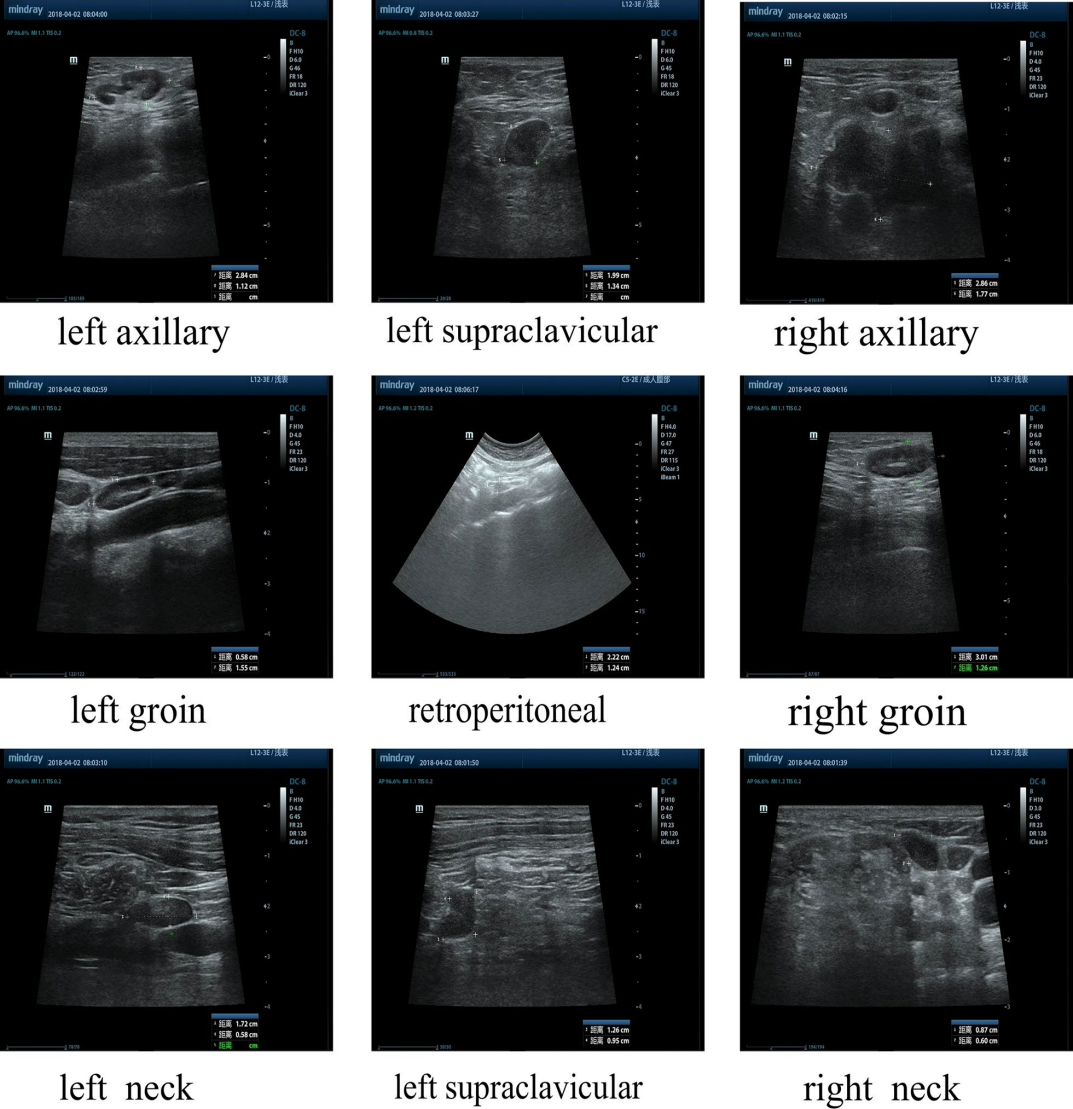
Ultrasonography showing enlarged lymph nodes bilaterally

**Fig. 2:**
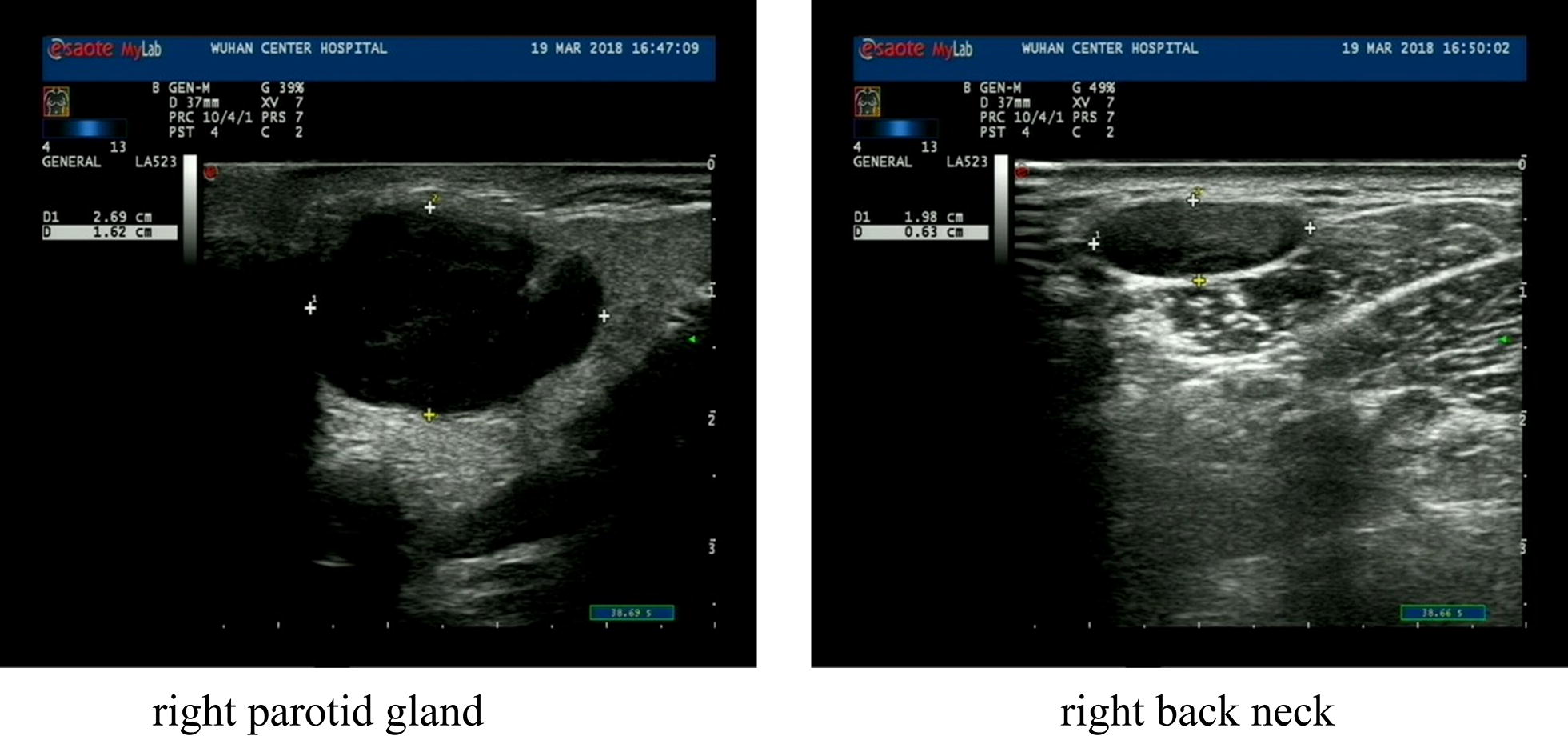
Ultrasonography showing enlarged lymph nodes in right parotid gland and back neck

Ultrasonography revealed the size of retroperitoneal (maximum 2.2 × 1.2 cm^2^), bilateral neck (maximum 1.7 × 0.6 cm^2^ on the left side, 0.8 × 0.6 cm^2^ on the right side), bilateral supraclavicular (maximum 2.0 × 1.3 cm^2^ on the left side, 1.3 × 1.0 cm^2^ on the right side), bilateral axillary (maximum 2.8 × 1.1 cm^2^ on the left side, 2.9 × 1.8 cm^2^ on the right side), and bilateral inguinal (maximum 3.0 × 1.3 cm^2^ on the right side) superficial lymph nodes. Enlargement of multiple lymph nodes was found in all areas (Fig. 1). Many solid nodules with a maximum size of 2.7 × 1.6 cm^2^ and 1.9 × 0.6 cm^2^ were found in the right parotid gland and right posterior neck, respectively (Fig. 2). Numerous solid nodules were seen on the left chest wall with a maximum size of 1.5 × 1.0 cm^2^. Pulmonary computed tomography (CT) scan showed obsolete pulmonary tuberculosis in the upper lobe of both lungs and localized dilatation of right upper lobe and middle lobe bronchi. Abdominal CT showed slightly high density of the left kidney.

Laboratory tests showed that the percentage of eosinophils in the patient’s whole blood was 39.40% (normal value is 0.4–8%), eosinophil count was 2.68 × 10^9^/L (normal value is 0.02–0.52 × 10^9^/L), and erythrocyte sedimentation rate was 47 mm/h (normal value is 0–20 mm/h). Blood sugar, urea, creatinine, and bilirubin levels and liver and kidney function biomarkers were within normal range. No abnormality was found in the routine urine test. Serum total immunoglobulin E (IgE) level was > 5000 kU/L (normal value: < 100 kU/L), serum specific IgE level to Phadiatop was 1.01 KUA/L (inhalation allergen screening, normal value: < 0.35 KUA/L), and serum specific IgE level to fx5 was 1.04 KUA/L (food combination, normal value: < 0.35 KUA/L), detected with ImmunoCAP (Phadia 250; Thermo Fisher Scientific Inc.). Allergen patch test was negative. Rheumatism, rheumatoid factor, anti-nuclear antibody, complete parasite set, routine stool and urine, thyroid function, immunoglobulin G4, complement C3/C4, and D-dimer levels were normal.

**Fig. 3:**
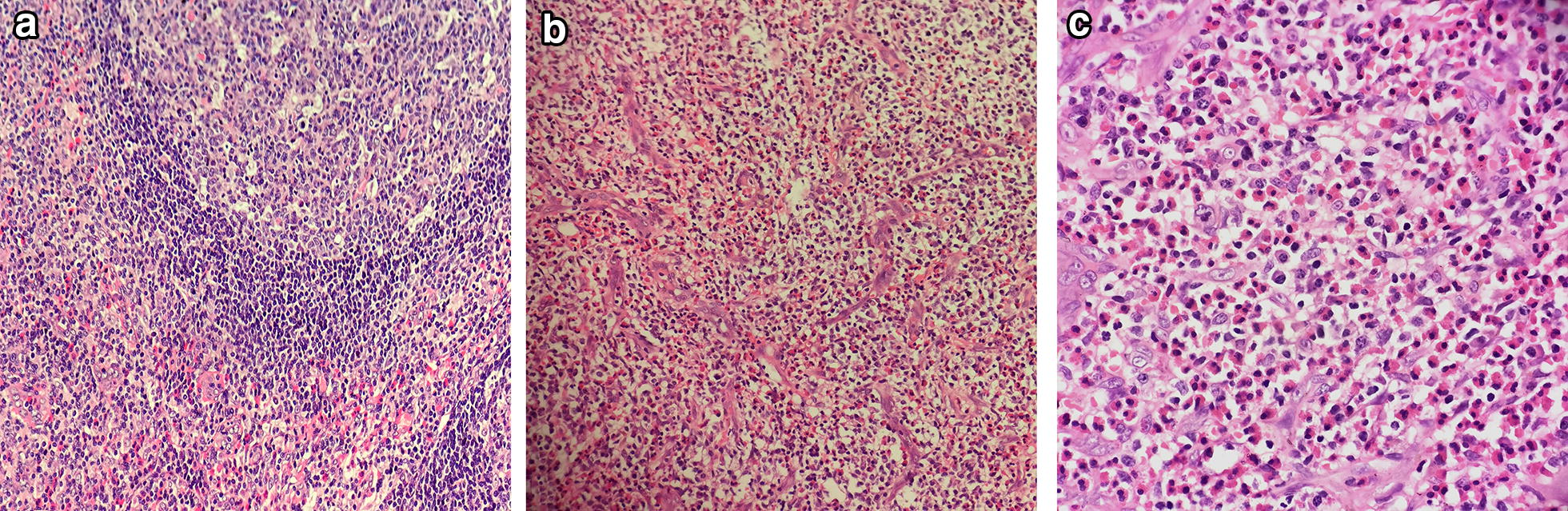
Histological features of excisional biopsy demonstrating eosinophilic infiltration. **a** Kimura Lymphadenopathy. Lymphoid follicle wth reactive germinal center (upper right), Intense eosinophilia with formation of eosinophilic microabscess(lower left). (H&E stain, ×100). **b** Same lymph node. Diffuse eosinophilia with capillary hyperplasia. (H&E stain, ×100). **c** Same lymph node. Eosinophilic microabscess formation with hyperplasia of endothelial cells in postcapillary venules. (H&E stain, ×400)

**Fig. 4:**
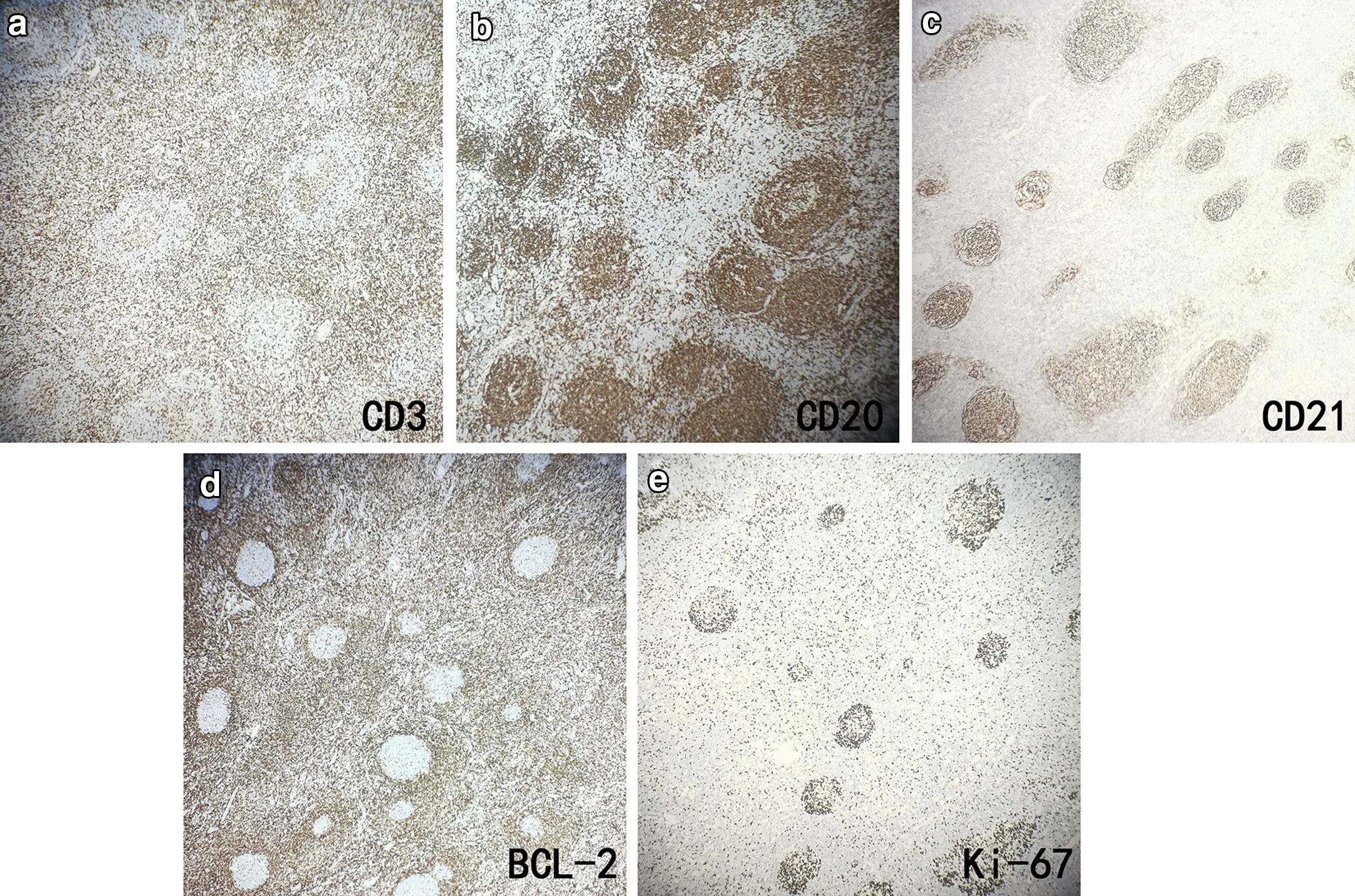
Immunohistochemical stains of excisional retroauricular lymph node shows reactive hyperplasia expression form. (EnVision two-step stain, ×40). **a** CD3 is expressed by numerous T cells located in interfollicular zone, fewer T cells in follicular germinal center, mostly of Th cells, **b** CD20 is expressed by abudantly hyperplastic reactive Lymphoid follicles, **c** CD21 highlights nodular FDC network. **d** BCL-2 stainning shows the germinal center of hyperplastic follices negative, but mantle zone of follicles and interfollicular zone positive, **e** Ki-67 stainning indicates the proliferation index of lymphocytes in germinal center of secondary follicular germinal centers high expression

After a complete examination, we resected the masses in the right neck, retroauricular and left axillary regions, and left chest wall directly. Postoperative pathological findings showed KD in the right neck, left chest wall, and left axillary lymph nodes (Fig. 3). Immunohistochemistry of the T lymphoid tissue suggested CD2, CD3, CD5, CD7, CD4, and CD8 positivity; BCL-6, CD10, and PD-1 negativity; no identified Reed-Sternberg (RS)-like cells (CD15 and CD30 negative) and proliferation of follicular dendritic cells (FDCs); and a nodular FDC network for CD21 (Fig. 4). The final diagnosis for this patient was KD. Low-dose radiotherapy may have been the best treatment, but she refused and only received glucocorticoid maintenance treatment after surgery; therefore, prednisone 60 mg QD orally was administered to the patient for 2 weeks and the dose was then gradually reduced over another 6 weeks. Lymph node enlargement ceased after 8 weeks of treatment. The patient is still taking prednisone 10 mg once a day, and the symptoms are well controlled even after 1 year.

## Discussion and conclusions

KD is characterized by eosinophilia in the peripheral blood and elevated serum IgE level. The extent of the lesion is closely related to the degree of eosinophil infiltration [5]. Typical manifestations of KD are solid, painless, or itchy single or multiple subcutaneous lesions on the head and/or neck, especially around the parotid gland and submandibular region, often associated with lymph node enlargement [6]. Up to 76% of patients with KD had lesions in the head and neck region [2], and although other regions such as axilla, groin, trunk, abdomen, peripheral limbs, chest wall, and median nerve have also been involved, their prevalence is extremely rarely [5]. The patient in this study manifested with solid nodules in the right neck and parotid region, accompanied by enlarged thoracic wall lymph nodes and axillary and inguinal lymph nodes. Pathological results confirmed KD. No studies have yet reported the occurrence of KD in multiple parts of the body at the same time.

Studies have shown that T cells are activated in the pathogenesis of KD, where they increase the permeability of the glomerular basement membrane, cause proteinuria, and even lead to nephrotic syndrome [7]. The incidence of nephrotic syndrome is approximately 19% in all patients with KD [8]. KD usually occurs prior to or along with kidney disease [8]. For the case in this study, the patient’s kidney function was normal and no proteinuria was observed in her urine. Only the abdominal CT showed a high-density image of the left kidney. It was not ruled out that her kidney might have been affected by the development of the disease, so close follow-up was recommended. A published case report also indicated that the clinical symptoms of KD are pruritus, as well as eczema and skin rash (although not common) [9]. This patient also had pruritus symptoms, presumably related to KD, but the specific mechanism was not clear.

Because of the painless enlargement of the lymph nodes in this patient, the diagnosis of lymphoma was considered first. In fact, KD is often misdiagnosed as a malignant hematological disease, such as chronic lymphoblastic leukemia or non-Hodgkin’s lymphoma, especially related to T cell lineage. CD30 staining, also known as Ki-1 or Ber-H2, can be used in the diagnosis of anaplastic large-cell lymphoma, classical Hodgkin lymphoma, and embryonic cancer prior to diagnosis of KD [10]. Immunohistochemistry results showed CD30 negativity and no RS-like cells, ruling out the possibility of lymphoma.

Ultrasound imaging is the primary examination for lymphadenopathy. KD lymph nodes are hypoechoic, solid, and round or oval; the surrounding soft tissues are normal [11]. Radiological examination can be used to determine the extent of the disease [12], but final diagnosis requires histological confirmation. Considering the possibility of lymphoma, we took a surgical biopsy in order to confirm the diagnosis for this patient as soon as possible and alleviate the symptoms. The histopathological features of KD are the formation of multiple lymphoid follicles with germinal centers, many of which are infiltrated by plasma cells, lymphocytes, mast cells, and especially eosinophils, which could eventually lead to follicular dissolution [13]. Histologically, KD usually involves eosinophil infiltration with follicular hyperplasia, fibrillar collagen hyperplasia, and dendritic vessel hyperplasia of the posterior capillary veins [14]. There is obvious eosinophil infiltration with occasional eosinophilic abscess formation [15]. The pathological features of the biopsy tissue were vascular lymphoid tissue hyperplasia with massive eosinophil infiltration, which is consistent with the pathological manifestations of KD.

In recent years, severe complications caused by KD, such as cerebral artery embolism, jugular vein embolism, pulmonary embolism, mesenteric embolism, and multiple arterial embolism in extremities have been reported [16]. It is speculated that eosinophilia may play a key role in the occurrence of embolism, but the specific mechanism is unclear [17]. In this case, the patient’s blood D-dimer result was negative, and the possibility of embolism was rare, but the patient was still advised to undergo reexamination and be alert to the risk of embolism.

Elevated eosinophil count and total IgE levels in peripheral blood are the most prominent features in the laboratory findings of KD, but specific IgE level was not clinically significant in the diagnosis of KD. Blood eosinophil count and total IgE levels, in particular, can help monitor the prognosis of KD, which is very important in its diagnosis and treatment [2]. Blood eosinophil count was significantly increased, and total IgE level was even higher than 5000 kU/L. However, special IgE levels for Phadiatop and fx5 were not high, which is consistent with the characteristics of KD in laboratory examination.

The etiology of KD is still currently unknown. Allergic reactions, *Candida* infection, arthropod bites, abnormal eosinophil dynamics, and IgE synthesis, as well as changes in systemic immune-mediated responses, are all considered to be the causative factors [18]. Various hypotheses have been proposed, including excessive proliferation of lymphocytes, release of interleukin-4 and interleukin-5 by mast cells, occurrence of eosinophilia and elevated IgE levels, and insufficient response to parasitic infections [19]. Most patients with KD are normally young and middle-aged males of Asian descent, and specific genes may also be involved in the pathogenesis of this disease [20]. It is very rare that the case we described here pertained to a Chinese Asian female patient.

The recurrence rate of KD is as high as 62% [21]. Studies have shown that eosinophil composition in peripheral blood exceeding 50%, total IgE levels in serum exceeding 1000 U/mL, and multiple lesions outside the salivary gland are the 3 main factors that may increase the likelihood of recurrence of KD [3]. As a highly relapsed disease, KD is very difficult to treat. At present, effective treatments include surgery, glucocorticoids and immunosuppressant, and low-dose radiotherapy [22]. Glucocorticoids can reduce blood eosinophil count and total IgE levels and play an important role in the treatment of KD, especially in patients with nephrotic syndrome [8]. Other recommended therapies are cyclosporine, imatinib, and cyclophosphamide [21]. Omalizumab is also available for patients with a serum total IgE level of < 1500 kU/L [23]. Studies have shown that [24] recurrence rate can be reduced to the lowest level after receiving low-dose radiotherapy. This patient refused to receive low-dose radiotherapy, so glucocorticoid had be given. Eight-week follow-up showed no lymph node enlargement. However, due to the high recurrence rate of KD and its occurrence in multiple parts of this patient’s body, and the high serum total IgE level which may promote disease recurrence, the patient should be informed to follow up closely.

To summarize, we reported the case of a 57-year-old Chinese female patient with KD affecting multiple body parts, which is extremely rare, and not consistent with the common affected areas and age characteristics of KD.

## Data Availability

Not applicable.
